# Systematic review on the prevalence of perinatal depression in Malawi

**DOI:** 10.4102/sajpsychiatry.v28i0.1859

**Published:** 2022-10-20

**Authors:** Genesis Chorwe-Sungani, Kondwani Wella, Patrick Mapulanga, Ditress Nyirongo, Mercy Pindani

**Affiliations:** 1 Department of Mental Health, School of Nursing, Kamuzu University of Health Sciences, Blantyre, Malawi; 2 Kamuzu University of Health Sciences Library, Lilongwe, Malawi; 3 Department of Mental Health, School of Nursing, Kamuzu University of Health Sciences, Lilongwe, Malawi

**Keywords:** perinatal depression, antenatal depression, postnatal depression, prevalence, Malawi

## Abstract

**Background:**

Perinatal depression causes significant burden to women and their families during the perinatal period. However, there is no reliable national prevalence data on perinatal depression in Malawi.

**Aim:**

This systematic review aimed at establishing the pooled prevalence of perinatal depression.

**Setting:**

The study setting is Malawi.

**Methods:**

Two reviewers conducted the search, selection, quality evaluation and data abstraction. Appropriate terms were used to search the CINAHL, PsychINFO, PubMed and ScienceDirect databases. The relevance and the quality of the studies were assessed. The prevalence of prenatal depression was pooled using a random-effects model, which was used to synthesise the data.

**Results:**

The review included a total of eight articles of fair and good quality. This review found a pooled prevalence of antenatal depression of 17.1% (95.0% confidence interval [CI]: 12.5–22.2) and postnatal depression of 19.8% (95.0% CI: 4.6–42.1) with an overall pooled prevalence of perinatal depression of 18.9% (95.0% CI: 14.5–23.8).

**Conclusion:**

This systematic review provided a pooled prevalence of perinatal depression which may be used in the absence of national prevalence data on perinatal depression.

**Contribution:**

This systematic review found a high a pooled prevalence of perinatal depression in Malawi suggesting that mental health should be a key component of maternal health programmes, policies and activities in the local setting.

## Introduction

Perinatal depression is a depressive mood disorder that affects women during pregnancy or in the first 12 months after the birth of a child.^1^ However, perinatal depression is often underdiagnosed by clinicians in Malawi.^2^ There is evidence that found a pooled prevalence of perinatal depression of 11.9% in women in lower-income countries.^3^ In Malawi, previous studies found that depression was prevalent during pregnancy (25.8%)^4^ and after childbirth (10.7%).^5^ This is within a range of prevalence rates for antenatal depression (8.3% – 41.0%)^6^ and postnatal depression (16.8%) in Africa.^7^ Therefore, Malawi constitutes some burden of perinatal depression in Africa.

There is no routine screening for perinatal depression in primary care settings in the country.^8^ Primary care is crucial in detecting, treating or making appropriate referrals to mental health care for women affected with perinatal disorders.^9^ It is also necessary to make efforts to identify risk factors for perinatal depression to assist in prevention, identification and treatment in primary care. Risk factors for perinatal depression include life stress, history of depression, maternal anxiety, lack of social support, lower frequency of exercise, unintended pregnancy, Medicaid insurance, intimate partner violence, history of child abuse, lower income, lower education, smoking, single status and poor relationship quality.^10^ This is corroborated by a study that found that lower perceived social support and intimate partner violence are associated with perinatal depression.^5^

Literature suggests that perinatal depression has a substantial impact on women during pregnancy^5^ and the first year after delivery. It is one of the major causes of disability among women during the perinatal period^11^ and can have serious long-term adverse effects on the well-being of women, their partners and infants.^10,12,13^ For instance, a systematic review found that some women who committed neonaticide, infanticide or filicide had mental health concerns.^14^ This may be the case in South Africa, where rates of neonaticide (19.6 per 100 000 live births) and infanticide (28.4 per 100 000 live births) are high.^15^ Furthermore, perinatal depression is linked to poor uptake of antenatal services,^16^ premature birth, intrauterine growth restriction, low birth weight,^17^ fatigue, poor concentration and feelings of hopelessness in a pregnant woman.^18^ Additionally, perinatal depression may affect mothers’ ability to provide sufficient nutritional care resulting in compromised infant growth and development.^19,20^

Perinatal depression may increase the risk of anxiety, depression, attention deficit hyperactivity disorder and conduct disorder in a child.^21^ Additionally, perinatal depression and human immunodeficiency virus (HIV) infection are linked in a vicious cycle, in which the symptoms of one disease exacerbate the other’s condition.^22^ Women who have co-morbid perinatal depression and HIV infection are less likely to adhere to antiretroviral medication, which is essential for their survival and the prevention of HIV transmission to their babies.^23^ This demonstrates the clinical and public health importance of perinatal depression^22,23,24,25^ in low-resource settings where the condition is highly prevalent.^24^ Although prevalence is often useful as it indicates the burden of a condition in a particular population,^26^ there is no reliable national prevalence data on perinatal depression in Malawi. Therefore, this systematic review aimed at establishing the pooled prevalence of perinatal depression in Malawi.

## Methods

The Preferred Reporting Items for Systematic Reviews and Meta-Analysis (PRISMA) guidelines were used to conduct the systematic review^27^ to address the review question.

### Search process

To find relevant terms in the title, abstract and subject descriptors, a limited search of PubMed and ScienceDirect was conducted. After that, search phrases and synonyms were identified for use in searching various databases for prevalence studies conducted in Malawi. No date limits were set, in anticipation that a wider period to be searched from the inception of each database will yield many relevant studies for possible inclusion in this systematic review. However, the search focused on articles that were written in English only. Search terms that were used are presented in Table 1.

**TABLE 1:** Search terms.

Database	Search terms used
PubMed	(((“perinatal”[All Fields] OR “perinatally”[All Fields] OR “perinatals”[All Fields] OR (“antenatal”[All Fields] OR “antenatally”[All Fields]) OR (“postnatal”[All Fields] OR “postnatally”[All Fields]) OR “antepartum”[All Fields] OR (“postpartum period”[MeSH Terms] OR (“postpartum”[All Fields] AND “period”[All Fields]) OR “postpartum period”[All Fields] OR “postpartum”[All Fields])) AND (“depressed”[All Fields] OR “depression”[MeSH Terms] OR “depression”[All Fields] OR “depressions”[All Fields] OR “depression s”[All Fields] OR “depressive disorder”[MeSH Terms] OR (“depressive”[All Fields] AND “disorder”[All Fields]) OR “depressive disorder”[All Fields] OR “depressivity”[All Fields] OR “depressive”[All Fields] OR “depressively”[All Fields] OR “depressiveness”[All Fields] OR “depressives”[All Fields])) OR (“depressive disorder”[MeSH Terms] OR (“depressive”[All Fields] AND “disorder”[All Fields]) OR “depressive disorder”[All Fields]) OR (“depression”[MeSH Terms] OR “depression”[All Fields] OR (“depressive”[All Fields] AND “symptoms”[All Fields]) OR “depressive symptoms”[All Fields])) AND (“epidemiology”[MeSH Subheading] OR “epidemiology”[All Fields] OR “prevalence”[All Fields] OR “prevalence”[MeSH Terms] OR “prevalence”[All Fields] OR “prevalences”[All Fields] OR “prevalence s”[All Fields] OR “prevalent”[All Fields] OR “prevalently”[All Fields] OR “prevalents”[All Fields]) AND (“malawi”[MeSH Terms] OR “malawi”[All Fields] OR “malawi s”[All Fields])))
CINAHL	TI prevalence AND TI depression OR depressive disorder OR depressive symptoms AND TI perinatal OR antenatal OR prenatal OR pregnancy OR postnatal OR postpartum OR Antepartum AND TI malawi AND LIMIT-TO (research article)
PsychINFO	Prevalence AND depression AND depressive disorder AND depressive symptoms AND perinatal AND antenatal AND pregnancy AND postnatal AND postpartum AND Antepartum AND prenatal AND Malawi
ScienceDirect	ALL (“depression” OR “depressive disorder” OR “depressive symptoms”) and ALL (prevalence AND pregnancy OR prenatal OR antenatal OR postnatal OR antepartum OR postpartum OR perinatal) AND LIMIT-TO (topics, “malawi”)

Using all identified search terms, electronic databases were searched for primary studies (PubMed, CINAHL, PsychINFO and ScienceDirect), and the results were imported into EndNote. Reference lists of key articles identified were hand-searched to identify additional articles. Manual searches of indexes and ‘grey’ literature databases were not carried out. The preliminary searches were conducted between July and August 2020, and the final search was done in October 2021.

### Data Collection

Upon completion of the search, duplicates and irrelevant articles (abstracts, conferences, congresses, editorials, commentaries, reviews, news and irrelevant records) were removed from the EndNote database, and later the search data was exported into Excel. The selection of articles for review was then conducted in three phases.

#### Abstract screening

This first phase involved reviewers independently scrutinising the titles and abstracts and indicating which articles were relevant in Excel. When the abstract did not provide enough information or the reviewers were undecided, the full-text articles were evaluated, and a consensus was reached between the reviewers on whether the article should be included or excluded. To determine the level of agreement for eligibility for inclusion at this step, a kappa statistic was calculated.

#### Screening based on participant, intervention, comparison, outcome and setting criteria

In the second phase of the selection process, articles were independently reviewed by two reviewers by applying and extracting the participant, intervention, comparison, outcome and setting (PICOS) criteria as follows: Participants (P) (pregnant women and mother of infants ≤ 12 months), Intervention (I) (any studies reporting the prevalence of perinatal depression), Outcome measures (O) (the measured prevalence of perinatal depression) and study setting (S) (Malawi). In addition, all primary quantitative studies that measured the prevalence of perinatal depression were considered for inclusion in the systematic review. Studies conducted outside Malawi were excluded.

#### Article review

Reviewers independently scrutinised full texts selected to confirm their inclusion by checking if an article reported the prevalence of perinatal depression in Malawi. Eligibility for full article review, assessment of study characteristics and relevant data extraction were assessed using a standard tool in Excel, and data were entered into a database. For each eligible study, the reviewers extracted information about the author, year of publication, title, setting, study design sample, assessment tool and prevalence rate of depression. Prevalence, measured based on a diagnostic assessment tool (Mini International Neuropsychiatric Interview [MINI] or Structured Clinical Interview for DSM [SCID]), was extracted from an article that reported multiple prevalence rates. All results were subject to double data entry.

### Assessment of methodological quality of studies

The critical appraisal tool for scoring methodological rigour of studies^28^ was used to assess the methodological quality of the included studies. However, the reliability and the validity of the tool are not documented in Malawi. The critical appraisal tool for scoring methodological rigour of studies was used in this systematic review to assess the quality of included studies.^28^ The tool has nine items that each assess the quality of a study on a scale as: very poor (1), poor (2), fair (3) and good (4). The tool has a minimum score of 9 and a maximum score of 36, and it allowed each reviewer to grade the included studies independently. Full texts of the included studies were retrieved and double-blind assessed for methodological quality by the two reviewers (P.M. and K.W.). The average score achieved by each study on the tool was used to determine its quality.

### Data analysis

Quantitative data analysis was conducted using MedCalc software.^29^ The calculated *I*^2^ Statistic (*I*^2^ = 91.9%, 95.0% confidence interval [CI]: 86.4–95.2) showed that the included studies were statistically homogeneous. However, because of clinical and methodological heterogeneity within and between studies,^30^ a random-effects model was utilised to calculate the pooled prevalence and 95% CIs. Pooling of prevalence and subgroup analysis by groups (antenatal depression and postnatal depression) were performed.

## Review process and results

**FIGURE 1:**
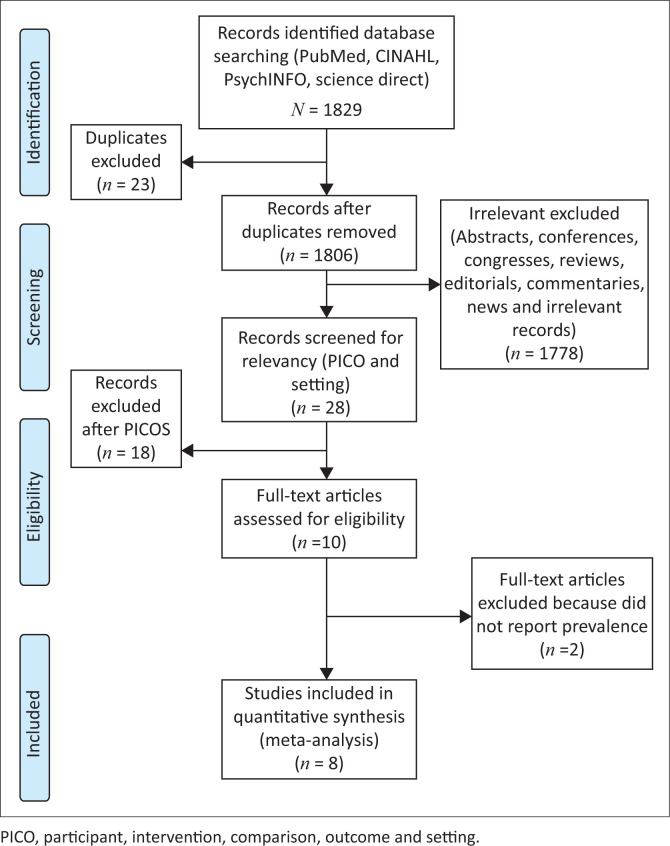
Study flow diagram based on Preferred Reporting Items for Systematic Reviews and Meta-Analysis (PRISMA).

The electronic search yielded 1829 published records from PubMed, CINAHL, PsychINFO and ScienceDirect (Figure 1). A total of 23 duplicates were removed, resulting in 1806 records that were scrutinised. A further 1778 records were removed because they were irrelevant (conferences, congresses, editorials, commentaries, reviews and news). The remaining 28 articles were assessed for relevancy by the reviewers using the PICOS criteria, excluding a further 18 articles, leaving 10 articles. The reviewers’ ratings for inclusion or exclusion of studies agreed with a kappa = 0.94. Of the 10 articles that remained, two were excluded^31,32^ because they did not report the prevalence of perinatal depression, resulting in eight studies that were included in this systematic review.

### The methodological quality of reviewed studies

All the eight included articles were rated for quality independently by G.C. and K.W. Overall, the quality was satisfactory with four articles^4,33,34,35^ rated as good, and four articles^5,20,36,37^ were fair (Table 2).

**TABLE 2:** Methodological quality of studies.

SN	Item	Study							
		LeMasters et al. (2020)	Chorwe-Sungani and Chipps (2018)	Chorwe-Sungani et al. (2021)	Dow et al. (2014)	Harrington et al. (2019)	Stewart et al. (2019)	Stewart et al. (2010)	Stewart et al. (2014)
1	Abstract and title: did they provide a clear description of the study?	4	4	4	4	4	4	4	4
2	Introduction and aims: was there a good background and clear statement of the aims of the research?	4	4	4	4	4	4	4	4
3	Method and data: is the method appropriate and clearly explained?	4	4	4	4	4	4	4	4
4	Sampling: was the sampling strategy appropriate to address the aims?	4	4	4	2	2	4	2	2
5	Data analysis: was the description of the data analysis sufficiently rigorous?	4	4	4	4	4	4	4	4
6	Ethics and bias: have ethical issues been addressed, and what has necessary ethical approval gained? Has the relationship between researchers and participants been adequately considered?	3	3	3	3	3	3	3	3
7	Findings or results: is there a clear statement of the findings?	4	4	4	4	4	4	4	4
8	Transferability or generalisability: are the findings of this study transferable (generalisable) to a wider population?	4	4	4	3	3	4	3	3
9	Implications and usefulness: how important are these findings to policy and practice?	4	4	4	4	4	4	4	4

	**Total**	**35**	**35**	**35**	**32**	**32**	**35**	**32**	**32**

	**Average score**	**4**	**4**	**4**	**3.6**	**3.6**	**4**	**3.6**	**3.6**

1 = very poor, 2 = poor, 3 = fair, 4 = good.

SN, serial number.

### Findings from studies for inclusion in the review (*n* = 8)

Of the eight articles that were included in this systematic review, seven articles were published in medical journals and one in a nursing journal, and their years of publication ranged from 2010 to 2021 (Table 1). Most of the articles were for cross-sectional studies (*n* = 6).

#### Instruments that were used to measure perinatal depression

The systematic review revealed that various instruments were used to assess for perinatal depression in Malawi (Table 3). The most-used instrument of the articles was Edinburgh Postnatal Depression Scale (*n* = 4), whose cut-off points ranged from ≥ 6 to >12. The second most-used instrument was SCID (*n* = 2), followed by the Self-Reporting Questionnaire 20 (*n* = 1), with a cut-off point ≥ 8, and the MINI (*n* = 1) (Table 3).

**TABLE 3:** Results for included studies (*n* = 8).

SN	Author	Year	Title	Setting	Design	Sample	Sampling method	instrument	Depression prevalence (%)
1	LeMaster et al.	2020	‘Pain in my heart’: Understanding perinatal depression among women living with HIV in Malawi	Malawi	Mixed methods	73 HIV positive pregnant women and postnatal mothers	Non-random sampling	EPDS cut-off ≥ 10	33.0
2	Chorwe-Sungani and Chipps	2018	A cross-sectional study of depression among women attending antenatal clinics in Blantyre district, Malawi	Malawi	Cross-sectional study	480 pregnant women	Simple random sampling	EPDS cut-off ≥ 10	19.0
3	Chorwe-Sungani et al.	2019	Validity of a two-question tool in detecting antenatal depression in Malawi	Malawi	Cross-sectional study	97 pregnant women	Simple random sampling	MINI	25.8
4	Dow et al.	2014	Postpartum depression and HIV infection among women in Malawi	Malawi	Longitudinal cohort study	529 HIV negative (156) and HIV positive postnatal mothers (373)	Nonrandom sampling	EPDS cut-off > 12	11.0
5	Harrington et al.	2019	Prevalence and factors associated with antenatal depressive symptoms among women enrolled in Option B+ antenatal HIV care in Malawi: A cross-sectional analysis	Malawi	Cross-sectional study	725 HIV positive pregnant women	Nonrandom sampling	EPDS cut-off ≥ 6	9.5
6	Stewart et al.	2019	Associations between antenatal depression and neonatal outcomes in Malawi	Malawi	Cross-sectional study	1391 pregnant women	Random sampling	SRQ 20 cut-off ≥ 8	14.2
7	Stewart et al.	2010	Common mental disorder and associated factors among women with young infants in rural Malawi	Malawi	Cross-sectional study	114 postnatal mothers	Random sampling	SCID	30.4
8	Stewart et al.	2014	A cross-sectional study of antenatal depression and associated factors in Malawi	Malawi	Cross-sectional study	583 pregnant women	Convenience sampling	SCID	21.1

EPDS, Edinburgh Postnatal Depression Scale; SCID, Structured Clinical Interview for DSM; MINI, Mini International Neuropsychiatric Interview; SRQ 20, Self-Reporting Questionnaire 20; SN, serial number.

#### Prevalence of perinatal depression in Malawi

The systematic review found that pooled prevalence of antenatal depression (17.1% [95.0% CI: 12.5–22.2]) (Table 4), postnatal depression (19.8% [95% CI: 4.6–42.1]) (Table 5) and the overall pooled prevalence of perinatal depression (18.9% [95.0% CI: 14.5–23.8]) (Table 6) were comparable. However, the pooled prevalence of postnatal depression was higher than the overall pooled prevalence.

**TABLE 4:** Pooled prevalence of antenatal depression in Malawi.

Study	Sample size	Proportion (%)	95% CI	Pooled effect estimate
Chorwe-Sungani and Chipps (2021)	97	25.8	17.4–35.7	14.9
Stewart et al. (2019)	1391	14.2	12.4–16.2	22.1
Chorwe-Sungani and Chipps (2018)	480	19	15.6–22.8	20.7
Harrington et al. (2019)	725	9.5	7.5–11.9	21.4
Stewart et al. (2014)	583	21.1	17.9–24.6	21.0
Pooled prevalence	3276	17.1	12.5–22.2	100.0

CI, confidence interval.

*I*^2^ = 91.8%, 95% CI: 83.7–95.8.

**TABLE 5:** Pooled prevalence of postnatal depression in Malawi.

Study	Sample size	Proportion (%)	95% CI	Pooled effect estimate
Dow et al. (2014)	529	11.0	8.4–13.9	51.4
Stewart et al. (2010)	114	30.7	22.4–40	48.6
Pooled prevalence	643	19.8	4.6–42.1	100.0

CI, confidence interval.

*I*^2^ = 95.8%, 95% CI: 87.9–98.5.

**TABLE 6:** Overall pooled prevalence of perinatal depression in Malawi.

Study	Sample size	Proportion (%)	95% CI	Pooled effect estimate
AND Chorwe-Sungani and Chipps (2019)	97	25.8	17.4–35.7	10.4
AND Stewart et al. (2019)	1391	14.2	12.4–16.2	14.2
AND Chorwe-Sungani and Chipps (2018)	480	19	15.6–22.8	13.6
AND Harrington et al. (2019)	725	9.5	7.5–11.9	13.9
AND Stewart et al. (2014)	583	21.1	17.9–24.7	13.7
PeND LeMasters et al. (2020)	73	32.9	22.3–44.9	9.5
PND Dow et al. (2014)	529	11	8.4–13.9	13.7
PND Stewart et al. (2010)	114	30.7	22.4–40	10.9
Pooled prevalence	3992	18.9	14.5–23.8	100

AND, antenatal depression; PeND, perinatal depression; PND, postnatal depression; CI, confidence interval.

*I*^2^ = 91.9%, 95% CI: 86.4–95.2.

## Discussion

Depression is the most common mental health condition that affects women worldwide during the perinatal period.^9,10,38,39,40^ It is necessary that women are screened for perinatal depression for early detection and treatment.^4^ However, the studies and instruments that are used to measure perinatal depression globally vary,^3,7,41,42^ leading to clinical heterogeneity. Clinical heterogeneity refers to variability in study population characteristics, interventions and outcomes across studies, while statistical heterogeneity includes methodological heterogeneity, biases and random error.^43^ This systematic review showed that the included primary studies had substantial clinical heterogeneity regarding study designs, sample sizes, sample characteristics and assessment tools. This might have affected the magnitude of the overall pooled prevalence that was generated in this study. As indicated by Kriston and colleagues, reviewers’ beliefs on the role of heterogeneity on the pooled prevalence found in this systematic review must be reflected.^44^

This systematic review suggests that the prevalence of perinatal depression in Malawi is high with an overall pooled prevalence of 18.9%. This pooled prevalence for perinatal depression in Malawi is lower compared to the one that was found in Ethiopia (25.8%).^45^ However, contrary evidence showed that the pooled prevalence of perinatal depression in Malawi is higher than that of women in lower-income countries (11.9%).^3^ This may be explained by literature which indicated that women encounter numerous risk factors during the perinatal period in Malawi.^4,5,46^ However, the need for conducting a proper national epidemiological study for perinatal depression still remains in the country. Epidemiological data will help in the implementation of targeted evidence-based interventions to protect the public^47^ and perinatal women in particular.

Literature suggests that violence, anxiety, life stress, prior depression and lack of social support are some of the risk factors for perinatal depression.^48^ Furthermore, prenatal depression is a risk factor for postnatal depression,^49^ so that pregnant women with untreated depression are more likely to suffer from postpartum depression and suicidality.^50^ This may be the case in Malawi, where this systematic review found that pooled prevalence for postnatal depression (19.8%) was higher than the pooled prevalence for antenatal depression (17.1%). Nonetheless, this result is contrary to the evidence that showed that pooled prevalence for postnatal depression (16.8%)^7^ was lower than the pooled prevalence of antenatal depression (26.3%)^42^ in Africa. Despite the variations, this review agrees with existing evidence that the prevalence of perinatal depression is high in Africa and Malawi in particular.

### Study limitations

This systematic review is limited in that there is a shortage of studies in Malawi to produce generalisability evidence. There is also a difference in the depression assessment tools, which may also have contributed to the detected heterogeneity in this review. Hence, caution should be taken during the interpretation of the results.

## Conclusion

This systematic review generated a pooled prevalence of perinatal depression for Malawi, which may be used by clinicians, researchers and policymakers in the absence of national prevalence data on perinatal depression. The review suggests that perinatal depression is highly prevalent in Malawi, based on a few published studies with inherently heterogeneous estimates. Based on this review, maternal health programme policies and activities should incorporate maternal mental health as a core component to promote early detection of perinatal depression and prompt interventions that would save the mother and her baby from different forms of morbidity and mortality.
